# Unlocking math potential in students from lower SES backgrounds – using instructional scaffolds to improve performance

**DOI:** 10.1038/s41539-025-00358-7

**Published:** 2025-09-23

**Authors:** Katharina M. Bach, Frank Reinhold, Sarah I. Hofer

**Affiliations:** 1 Ludwig-Maximilians-Universität München, Munich, Germany; 2 Pädagogische Hochschule Freiburg, Freiburg, Germany

**Keywords:** Psychology, Education

## Abstract

Socioeconomic status (SES) influences school success. Students with lower SES may face challenges that this study aims to address through instructional scaffolding. To be effective, such support needs to consider students’ individual strengths and weaknesses. In this study, 321 sixth-grade students used an e-textbook about fractions. They were randomly assigned to receive either adaptive task difficulty, explanatory feedback, or dynamic visualizations as scaffolds or no scaffolding. We assessed their fraction knowledge at pre- and post-test and eigth cognitive and motivational-affective characteristics. Latent profile analyses identified three profiles. Students with lower SES (below the nationwide average) are commonly associated with a profile that has unfavorable learning prerequisites. A linear mixed model revealed that adaptive task difficulty significantly benefited students in this profile. Implementing adaptive task difficulty in math classes might mitigate challenges associated with lower SES, enhancing educational success and equity by addressing individual prerequisites and learning needs.

## Introduction

Socioeconomic status (SES) is strongly associated with students’ learning and performance^1,2^. Several meta-analyses emphasized the role of lower family SES as a risk factor for educational achievement^3–5^, accounting for up to one-third of the disparity in educational outcomes^6^. SES encompasses a person’s economic and social status and can be measured via education, occupation, or income^7^. Since it is operationalized and measured differently, there are no universal cut-off values for low and high SES. Therefore, we tend to assume trends in this study and only refer to comparatively higher and lower SES.

Students growing up in lower SES families often exhibit impaired performance and lower grades than students from higher SES families^8,9^, resulting in critical achievement differences irrespective of the subject^10,11^. Recent panel results, such as those from PISA, support these findings robustly^12–14^.

The influence of SES and the differences in opportunities and achievements between students from lower and higher SES backgrounds are particularly pronounced in math education^15–17^. PISA test scores of 41 countries indicated that students with a higher SES reached higher math scores than those with a lower SES^18^. PISA results from Germany demonstrated that around half of the students from lower SES backgrounds performed below the baseline level, reflecting a lack of basic math skills^19^. Such results indicate potential challenges that students from lower SES backgrounds may face in the future^19^, given the crucial role of math competence in gaining degrees and finding employment in high-skill workplaces^20,21^.

SES does not influence achievement directly, but as a formative construct, it can alter life circumstances and opportunities, affecting achievement. Students from lower SES families often enter schools with prerequisites different from those of their peers. Often, these students have less favorable home learning environments^22,23^ with little or no support, education-enhancing parenting practices, and stimulating activities outside the school^24,25^. Parents’ time- and energy-consuming occupations (e.g., shiftwork)^26^ and psychological stress caused by the economic strains^27^ might limit their capacity to support their children’s educational endeavors. Moreover, limited financial resources restrict access to external resources like private tutoring^28^, books, learning materials^29^, or necessary technical equipment and software^30,31^. Moreover, external influences that devalue students from lower SES backgrounds can contribute to a weaker performance: A prevailing stereotypical narrative often portrays them as incapable of achieving academic success. Internalizing these prejudices might lead to casting doubt on their abilities and feeling threatened by the associated stereotypes^32,33^. These feelings can pose an immense cognitive and emotional burden^34^, further hindering their performance^35^.

### Influence of learners’ characteristics on academic performance in math and their relationship with SES

Understanding how SES affects characteristics relevant to learning and how to address these characteristics through targeted support is essential. Therefore, we consider a non-exhaustive selection of students’ cognitive (prior knowledge, sustained attention, visuo-spatial ability, and reasoning skills) and motivational-affective (engagement, self-concept, interest in math, math anxiety, and excessive demand) learning prerequisites.

We assume that students’ level of performance and their cognitive and motivational-affective learning prerequisites are not a direct consequence of inherent abilities and characteristics but are strongly influenced by students’ living conditions, their home learning environments, and family support, which may limit the potential for development. It might be possible to mitigate the symptoms caused by, for example, a lack of support at home within the classroom through targeted interventions.

#### The relation between SES and cognitive characteristics

##### Prior knowledge

Prior knowledge is considered a central predictor of learning and performance, facilitating problem-solving^36,37^. Domain- or content-specific knowledge plays a crucial role in academic success^38^, for example, in math^39^. As described above, students from lower SES backgrounds often exhibit poorer math performance^40^. The reasons and mechanisms for this are discussed in the following paragraphs by looking at other cognitive and motivational-affective characteristics.

##### Sustained attention

Sustaining attention means maintaining focus on a specific task for an extended period despite distractions^41,42^ which positively predicts academic achievement, particularly in math^43,44^. Conversely, low-achieving students tend to exhibit shorter attention spans and greater susceptibility to distractions^45^. Sustained attention mediates family characteristics, particularly family SES, and academic achievement^46^. From an early age, children from lower SES backgrounds are more likely to display difficulties in selective and sustained attention^47,48,49^ and have a higher prevalence of attention deficit hyperactivity disorder^50^. These disparities might be attributed to differences in the home learning environments, including the availability of books and toys, as well as to the interactions and (cognitive) activities of parents and children requiring sustained attention, such as reading together^51,52^. Moreover, the relationship between adverse childhood events (ACE; such as parental divorce), attention disorders, and SES is noteworthy. Students from lower SES backgrounds have a higher incidence rate of such ACEs, which can increase their risk of attention difficulties^53,54^.

##### Visuo-spatial ability

Visuo-spatial ability describes an individual’s ability for “generation, retention, retrieval, and transformation of visuo-spatial information”^55^ (p. 903). It is strongly linked to math performance, particularly in visuo-spatial tasks (e.g., mental rotation)^56–59^. Notably, differences in students’ visuo-spatial ability are associated with differences in their SES^60,61^ (medium effect^62^). The lack of access to activities and engagement with particular toys like Legos, motor toys, puzzles, or specific video games fostering the development of visuo-spatial skills^63–66^, might explain why students from lower SES backgrounds often have underdeveloped visuo-spatial ability^65,67^.

##### Reasoning skills

Non-verbal relational reasoning, a subset of intelligence^68^, encompasses the ability to identify repeated patterns, logical sequences, or classifications without relying on language or verbal cues^69^. It predicts math performance^70,71^, particularly in standardized tests (correlations up to .90 in TIMMS and PISA)^72,73^. As a broader concept, intelligence is related to family characteristics, including SES^74–76^. Past research revealed significant disparities between students from lower- and higher-SES families^77^. The heritability of intelligence that is mentioned in some research^78,79^ varies as a function of SES^80^: For students from lower SES backgrounds, environmental factors such as the living environment and parental support play a more crucial role than genetic factors^76,81–83^. As such, the potential lack of educational stimulation and support in their early childhood environments^84,85^ may hinder many of these students from realizing their inherited cognitive potential^86,87^. Especially parents’ education, a component of SES, often shapes children’s relation to education and the support they receive^88,89^. Additionally, nutrition, which often varies between lower- and higher-SES families, can impact intelligence development during childhood^90^. Specialized early childhood intervention programs in the U.S. have successfully narrowed income-based cognitive gaps, supporting the idea that the influence of SES on intelligence is more linked to unfavorable home learning environments, little familiar support, and a lack of remediation than heredity^91,92^.

#### The relation between SES and motivational-affective characteristics

##### Cognitive and behavioral engagement

Engagement refers to active involvement and students’ focus on, for example, a class activity^93,94^. It is typically conceptualized as a two-dimensional construct^95^: First, behavioral engagement includes “positive conduct, effort, [and] participation”^96^(p. 429) and primarily describes students’ observable behaviors in the classroom (e.g., hand-raising)^97^. Second, cognitive engagement pertains to cognitive strategies (e.g., learning goals) students employ, their will to master knowledge, and the extent to which they are cognitively involved during a task^93^. Both forms of engagement positively impact academic achievement, while their absence can result in underperformance and failure at school^98,99^. Engagement is considered a mediator between family context and educational outcomes^100^. Students with higher SES demonstrate higher school engagement than their peers from lower SES backgrounds, leading to poorer performance^101,102^. These differences may be attributed to the lower value placed on education within families with lower SES, potentially leading to reduced engagement in school and absenteeism^102^. Additionally, the stress these students experience due to their financial hardship and associated physical and psychosocial stressors^103^ might impair cognitive functions^104^, making it more challenging to be engaged in class.

##### Self-concept

The self-concept refers to an individual’s perception of themselves^105^ in different contexts^106^. The academic self-concept pertains explicitly to the context of schools and universities^107^ and academic performance^108^. In math, the academic self-concept is among the strongest predictors of students’ grades^72^. However, the relationship between academic performance and self-concept is reciprocal^109^. On average, students with higher SES report a higher academic self-concept than those with lower or medium SES^110^. The general math and science self-concept can be influenced by home environment factors such as internet access and computer availability, which are affected by household income^111^. In addition, negative stigmas and stereotypes^112^ can be internalized and, thus, might negatively affect an individual’s self-concept^113,114^. Moreover, in competitive school environments, students from lower SES backgrounds tend to engage in upward comparisons with their peers, further harming their self-concept^115^.

##### Interest in math

Interest in a particular topic or subject motivates individuals to deepen their understanding, which is essential in education^116^. Interested students spend more time with the topic and tend to derive more enjoyment from it^117^, which can enhance their performance^118^. The relationship between interest and math performance appears to be reciprocal^119,120^. Findings on how interest varies based on SES are conflicting: There is research suggestig that students from lower SES backgrounds show more interest in math and education in general than students with higher SES and similar skill levels^121^. Other results indicated the that students from families with higher SES are more interested in math than their peers from lower SES backgrounds because they often experience higher levels of success in the subject^122^ and students generally tend to show more interest in subjects in which they excel^123,124^.

##### Math anxiety

Some students experience anxiety before and during math class^125^, which can hinder their performance in this subject^126,127^. Math-anxious students tend to avoid math classes, resulting in fewer practice opportunities^128^. Furthermore, anxiety can overload the working memory. For example, intrusive thoughts about a perceived lack of skills hinder processing math tasks (Ashcraft et al., 1999 cited in^129^). Since performance difficulties can also contribute to higher math anxiety, a reciprocal relationship between math achievement and anxiety can be assumed^130^. Lower SES is a risk factor for math anxiety^131^. Parents’ attitudes toward math may influence their children^132^: Parents with lower SES and sometimes less access to formal education may show aversion towards math, which could exacerbate math anxiety in their children^133^. The negative stigma associated with lower SES^112^ can impact math anxiety, as seen, for example, in Black and Latinx students in the U.S.^134^.

##### Excessive demand

We define excessive demand as an individual’s (mental) effort needed to solve a specific task. High excessive demand is associated with feeling overwhelmed and having less confidence in one’s ability to solve a task^23^. As such, this construct represents a counterpart to self-efficacy^135^. Learners with high excessive demand might be overwhelmed by the workload and difficulty level. The level of excessive demand depends not only on the task per se but also on the students’ familiarity with the content and the type of task^136^, which enhances students’ confidence^137^. Assuming that students from lower SES backgrounds potentially have less exposure to learning tools and games, they might be less familiar with specific types of math tasks^138^, which might overwhelm them. Additionally, students who have already experienced failure in math have lower self-efficacy^139^. This influence seems to be more pronounced for marginalized than for more privileged students^140^. Correspondingly, students from lower SES backgrounds may experience higher excessive demand in school^141^.

### Fostering educational equity through scaffolding

Targeted instructional support, addressing relevant cognitive and motivational-affective characteristics, might mitigate the potentially multifaceted mechanisms of how lower SES can impair math performance. The interventions should foster students’ existing resources and, at the same time, focus precisely on the areas where students have specific shortcomings. Scaffolding^142^ is a form of instructional support to guide students in their learning and problem-solving^143–145^. Through effective scaffolding that can take different forms (e.g., feedback or dynamic visualizations), students can complete tasks and reach performance levels that would be impossible without support^145–147^. However, scaffolding is not equally beneficial for all students but depends on their prerequisites comprising cognitive, metacognitive, and motivational-affective characteristics^142,148,149^.

Cognitive and motivational scaffolds are considered two major types of instructional scaffoldings^113^, next to metacognitive scaffolding^150^. Cognitive scaffolds provide learners with additional information (information scaffolds) or activate prior knowledge to support them in their information processing (information processing scaffolds). Motivational scaffolds mainly impact learners’ motivation and commitment. Therefore, considering students’ characteristics^151–153^ to determine the most effective scaffolds is a promising way to combat the challenges associated with lower SES and promote educational equity. It is important to note that the classifications of motivational, informational, and information-processing scaffolds are not deterministic but rather reflect the intended purpose of the scaffolding^154^. Specifically, they address the unfavorable individual characteristics that the scaffold compensates for. Consequently, the same type of scaffold can have several intentions and fulfill different functions within students’ learning processes. The classification of a scaffold depends on the intention (e.g., compensating for insufficient motivational-affective characteristics would classify it as a motivational scaffold) and the theoretical foundation, which allows for varied justifications.

In the present study, we implemented three examples of instructional scaffolding that can be classified these three scaffolding intentions.

#### Adaptive task difficulty

Adaptive task difficulty is classified as a form of motivational scaffolding supporting students’ practice^113^. During practice, the difficulty level is continuously adjusted to match the learners’ performance levels, which are constantly monitored^155^. Adaptive scaffolding aims to offer an appropriate level of challenge aligning with students’ zones of proximal development^156^, ensuring it neither over- nor under-challenges the learner^157^. Adaptive instruction has positively affected students’ learning experiences and performances across various subjects, including math^158–160^. A meta-analysis has also shown that adaptive changes in difficulty levels are more effective than fixed, incremental increases^161^. The benefits of adaptive task difficulty primarily relate to motivation and affect. Learning at a suitable difficulty level helps alleviate anxiety^151^ and can improve attitudes toward challenging subjects such as math^162^. Learning at their own pace and a suitable difficulty level fosters a sense of accomplishment, creates positive learning experiences, and increases students’ motivation and interest^159,163^. Given the positive affective effects on low-achieving students^151,164^, adaptive task difficulty can be expected to be especially beneficial for students from lower SES backgrounds who often display unfavorable values on the motivational-affective characteristics, such as high math anxiety^131^ and low academic self-concept^111^.

#### Explanatory feedback

Feedback, classified as a form of information scaffolding^113^, is most effective if it extends beyond merely evaluating the correctness of students’ solutions^165^. Explanatory feedback has a more significant effect on learning outcomes than corrective feedback, as a meta-analysis shows^166^. Receiving immediate feedback that elucidates the correct approach to the given problem, increases future performance^167^. This form of feedback proves effective by reducing the learners’ cognitive load and helping them construct knowledge^168^. As such, explanatory feedback is a crucial feature of technology-enhanced learning platforms, especially for independent student work^168^. Students with low prior knowledge can benefit from explicit information on how to solve the problem^169^. However, they need adequate cognitive resources (e.g., working memory capacity) to use the feedback^170^. As students from lower SES backgrounds are likely to be among those with lower prior knowledge^40^, explanatory feedback is considered one of the most effective interventions for students from lower SES backgrounds^81^.

#### Dynamic visualizations

Dynamic visualizations are classified as an information-processing scaffold^113^ that supports students in grasping new information. Technology-enhanced learning environments offer interactive experiences that allow learners to manipulate different parameters within such dynamic visualizations or simulations (e.g., change the representation)^171^. For example, students can engage with interactive diagrams that change their representation based on the students’ clicks^142,172^. One aim of dynamic visualizations is to support students in learning with analogies^173^ and integrating prior knowledge with new information^174^. Research results regarding the effectiveness of dynamic visualizations compared to static ones are mixed^175^. Yet, some recent reviews suggest dynamic ones may be more effective^176,177^.

Studies on who benefits most from dynamic visualizations show contradictory findings^113,178,179^. The differences in findings may depend on the complexity of the content and the visualization style. Dynamic visualizations of easy topics, displayed in schematized visualizations, are particularly helpful for individuals with low visuo-spatial ability and those of complex topics represented in realistic visualizations are beneficial for individuals with high visuo-spatial ability^113,180^. Since students from lower SES backgrounds are often associated with low visuo-spatial ability^61^, they might particularly benefit from dynamic visualizations for less complex topics.

### Present study

In the present study, we examined the effects of lower SES, operationalized through parental occupations, on various cognitive and motivational-affective characteristics associated with academic performance. Precisely, we aimed to identify typical profiles of students from lower SES backgrounds. To achieve this, we focus on a non-exhaustive selection of variables central to learning, related to SES, and targeted by the three types of instructional scaffolding. Most studies to date have focused on individual variables alone, rather than comprehensive profiles that incorporate multiple variables. However, a variable-centered approach might overlook the resources of students with lower SES backgrounds. Using person-centered profile analyses, we try to comprehensively identify systematically occurring combinations of individual characteristics of students from lower SES backgrounds. Moreover, the person-centered approach allows us to comprehensively map how students may exhibit favorable scores in some areas and unfavorable ones in others. Based on these individual profiles, we sought to determine which of the three types of instructional scaffolds would enhance their knowledge of fractions on the number line best. The number line is a common model used to represent fractions in math instruction^181^. Moreover, fraction understanding, included in curricula across school types, is a basic math skill relevant for further math education and daily life^182–185^.

In our sample, we include all students, regardless of their SES, and cluster them later based on the analyses, which include SES. This also allows us to compare students from lower and higher SES, even though it is not the primary aim of our study. Based on the research findings described above, we hypothesized that the profiles of students from lower SES backgrounds compared to those of higher-SES students are overall likely to be characterized by lower scores on cognitive characteristics (i.e., lower prior knowledge, general reasoning, visuo-spatial attention, and sustained attention) and on motivational-affective characteristics (i.e., lower engagement, interest in math, and self-concept) and higher scores on math anxiety and excessive demand. Nevertheless, students from lower SES backgrounds also differ significantly in their home learning environments and familiar support, potentially leading to differences in their learning prerequisites. Profile analyses allow for detecting such differences in the combination of individual characteristics among students from lower SES backgrounds. Given the variability in potential profile compositions arising from the nine characteristics, we could not anticipate the specific profiles that would emerge from our analyses (e.g., composition and number of profiles) and how the profiles would ultimately represent the students from lower SES backgrounds. So, we aim to answer the following research question:

RQ1: How can subgroups of students from lower SES backgrounds be described in terms of cognitive and motivational-affective characteristics?

Building on the results of RQ1, we aimed to explore the differential effectiveness of three types of scaffolding with different intentions, particularly focusing on the profiles associated with lower SES. We expected that adaptive task difficulty positively affects students with unfavorable scores on the motivational-affective variables. Moreover, we expected explanatory feedback to positively impact students with lower scores on the cognitive variables. Lastly, we predicted that students with lower visuo-spatial skills would benefit from dynamic visualizations in the context of fraction learning. We did not expect any significant effects of the instructional scaffolds for students with favorable characteristics of the corresponding variables, nor significant main effects for the entire sample.

Accordingly, we aim to address the following second research question:

RQ2: How do students in the profiles associated with lower SES benefit from three different types of cognitive and motivational scaffolds (i.e., adaptive task difficulty, explanatory feedback, and dynamic visualization) compared to no scaffold in terms of their fraction understanding?

With our two research questions, we combine two research traditions: higher-level research on SES and origin effects and research on micro-level learning and scaffolding processes. This connection of two perspective is worthwhile, as learning always takes place in a larger context, which in combination with individual characteristics has an impact on micro-level learning processes.

## Methods

### Participants

Data came from 338 German sixth-grade students. After excluding participants without data on SES, one of the most central variables of our study, the final sample was reduced to *N* = 321 (*n*_female_ = 142, *n*_male_ = 173, *n*_other/prefer not to say_ = 5, and missing information for one student). We decided to exclude these students without data on SES despite the risk of introducing a potential bias as we cannot be sure that the data are missing at random. Particularly in the case of data on parental occupation as sensitive data, it is possible that students did not provide any information due to shame or other personal reasons and that the 17 students are potentially from a specific group (e.g., with lower SES). Thus, due to the sensitivity of the data and the small proportion of the total sample (5.03%), we decided not to impute any data.

In this sample, 131 students attended the non-academic track schools (*Mittelschule)* and 190 the academic track schools (*Gymnasium*). They came from 16 classrooms at three non-academic and one academic track school. All students from the academic track schools were recruited in 2021, while students from non-academic track schools were recruited in both 2021 (*n* = 55) and 2022 (*n* = 76).

Germany has a tripartite secondary school system. Students typically transfer from elementary school to different types of secondary schools after fourth grade^186^. Non-academic and academic track schools differ primarily in their orientation and the length of schooling until graduation. Non-academic track schools (graduation after ninth or tenth grade) focus on vocational apprenticeships and are considered the less demanding tracks, while academic track schools (graduation after 13^th^ grade) prepare students for higher education^187^. Only if they obtained the school-leaving certificate from academic track schools, students are allowed to enter university. The different types of schools also differ in their learning environments, curricula, and teaching cultures^188^. Past research has indicated that students at academic track schools are often advantaged and have better chances to develop their performance compared to their peers at non-academic track schools^189^. One often-discussed reason for this is that the two types of schools differ in their student composition, with typically more students at risk, including those with lower SES or migration history, attending non-academic track schools^188,190^. Nevertheless, for both types of schools, fractions are included in the Bavarian curriculum in sixth grade. So, students were formally introduced to fractions before participating in our study but not to fractions on the number line, which the present study focuses on.

### Procedure and intervention conditions

The cross-sectional study was conducted as part of a larger research project, a collaboration between the Ludwig-Maximilians-Universität Munich and the Pädagogische Hochschule Freiburg. The project is based on ALICE, developed at the Technische Universität Munich (www.alice.edu.tum.de). ALICE is a digital learning environment that was developed as part of a cooperation between the Chair of Geometry and Visualization at the Department of Mathematics and the Heinz Nixdorf Foundation Chair of Mathematics Education, both at the Technische Universität Munich, and was adapted by Hofer's team for the current project. The project was reviewed and approved by the Bavarian Ministry of Education, Germany (reference: IV.7-BO4106.2019/52/9). This authorization includes ethics approval. The present study was conducted at two independent measurement points at the end of the school years 2021 and 2022, respectively. The pre- and post-tests, as well as the intervention, were always carried out on the same day.

Following the approvals from principals and teachers, parents’ informed consent for students’ participation was obtained. Students could participate regardless of their SES. They were randomly assigned to one of three intervention groups with different instructional scaffolds (adaptive task difficulty, dynamic visualization, and explanatory feedback) or the control group, which worked through the same materials but did not receive additional scaffolding. Approximately the same number of students were allocated to each condition within each participating class. Students participated with the tablets provided by the researchers. The two-hour assessment took place in the classroom during regular school days, guided by an investigator. The students worked independently, and neither the investigator nor the teacher provided any additional support beyond the general instructions and the support available in the e-textbook.

**Fig. 1:**
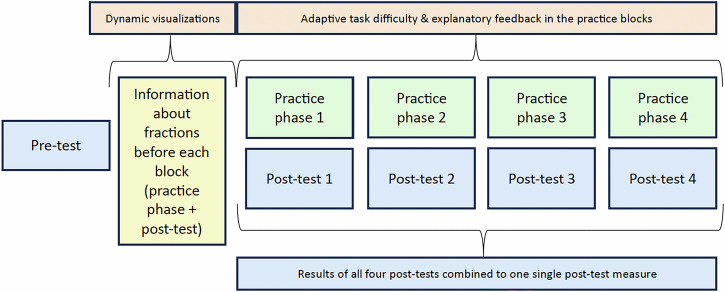
Scaffolds apply only to the associated intervention group, not to everyone.

First, students received a short declarative input regarding fractions before they conducted the pretest, which consisted of four parts of increasing difficulty, assessing students’ fractions on number line knowledge. Afterward, they proceeded with the intervention, which consisted of four blocks of content and tasks of increasing complexity in an e-textbook. The increasing complexity was based on specific characteristics of the number line, such as its length (i.e., length between 0 and 1 or longer) and equivalence (i.e., the number line is segmented according to the value of the denominator of the fraction that needs to be placed or not). Each block began with written information complemented with static visualizations of fractions on number lines (see Supplementary Fig. 1). Students in the dynamic visualization condition were provided with interactive dynamic visualizations, allowing them to modify the division of the number line (see Supplementary Fig. 2). Next, students in all conditions summarized their ideas in an open-response format, facilitated with two reflection questions (see Supplementary Fig. 3). Afterward, they practiced placing fractions correctly on a number line for five minutes (see Supplementary Fig. 4). No specific number of tasks had to be completed; instead, the students could complete as many tasks as they chose. During the practice phase, the difficulty level was adjusted to the students’ performance in the adaptive task difficulty condition based on empirically validated difficulty-generating factors^191^: a random set of five tasks was generated on the pre-defined set of difficulty generating factors; students solving three or fewer of these tasks received another set within the same difficulty level (i.e., with exactly the same difficulty generating factors present); students solving more than three tasks correctly received a set with increased difficulty. In all other groups, difficulty increased after each set independently of students’ performance. After each task, students received corrective feedback telling them whether their answer was correct and, if not, providing them with the correct solution (Supplementary Fig. 5). In the explanatory feedback condition, students received explanatory feedback, including information on their mistake (by analyzing their input and the resolving most-likely error strategy^191^) and on ways to reach the right solution (Supplementary Fig. 6). For more in-depth insights into the design of the specific unit and tasks of the e-textbook, see refs. ^113,164^. Following this practice phase, a block-specific post-test assessed the students’ fractions on number line knowledge without any feedback on their answers. The procedure is illustrated in Fig. 1.

### Procedure of the intervention

This procedure was repeated for all four blocks, which increased in complexity due to elements present in the tasks. In the introduction before the intervention, the denominator of the fraction fitted to the division of the number line of length 1; in block 1 (the first intervention block), the division of the fraction had to be altered (by expanding and reducing the fraction) to place it on the number line; in block 2 the length of the number line exceeded 1 additionally; in block 3 ticks on the number line were missing in addition; in block 4 fractions had to be placed on an empty number line.

Finally, various individual characteristics and demographic information, including SES, were collected via an online questionnaire (Lime Survey).

### Instruments and scales

#### Socioeconomic status

Students’ SES was assessed using the International Standard Classification of Occupations (ISCO-88)^192^ and International Socio-Economic Index (ISEI)^193,194^ based on self-reports of their parents’ occupations, which is considered one of the three most popular measures of SES^195^. We asked students for their mother’s and father’s professions and job activities (i.e., “What does your mother/father do in their job”?). Their answers were then coded with the ISCO-88 codes that comprise over 400 different occupations that are grouped into 28 areas. The ISCO-88 codes were then converted into the ISEI scores that can take values between 16 (cleaners and agricultural assistants) to 90 (judges). Additionally, we added a score of 0 for unemployment. The scores were created for both parents (if applicable), and the higher ISEI score was used to define students’ family SES (HISEI). This procedure is common to measure SES and is also used in PISA studies^196^.

#### Cognitive and motivational-affective characteristics

Table 1 provides detailed information about all instruments and scales used to assess the cognitive and motivational-affective characteristics. All characteristics except the visuo-spatial ability (paper-based test) were assessed via tablets using established tests.

**Table 1:** Overview of all instruments and scales used to assess cognitive and motivational-affective characteristics

Characteristics	Operationalization	Adapted from	Sample items
*Cognitive characteristics*			
Sustained attention	For three minutes, participants have to respond to stimuli (pictorial flowers) and sort them up or down, based on two memorized rules. The number of z-standardized hits, omissions, mistakes, and dismissals is collected. A score is built by subtracting the number of mistakes and omissions from the hits. (score ranging between −1 and 1)	Attention Swiping Task (AST)^245^	See Supplementary Fig. 7
General reasoning skills	As in classical matrices, nine fields are shown, each following a specific rule. Following this rule, students must fill in the ninth field by composing their answers from a selection of 20 elements. Students have 16 min to complete 16 matrices task. (maximum score = 16)	DESIGMA construction-based figural matrices task^246^	See Supplementary Fig. 8
Visuo-spatial skills	In a total of five tasks, students are presented with a paper folding template and need to choose from a selection of five objects, the one that can be folded out of this. (maximum score = 5)	Paper-Folding-Test of the Berliner Intelligenz-Struktur-Test (BIS-Test)^247^	See Supplementary Fig. 9
*Motivational-affective characteristics*			
Math anxiety	The five items allow mapping different aspects of math anxiety, such as feeling worried, helpless, or nervous during or concerning math classes and problems.	ANXMAT scale, used in the PISA assessments^248^	“I feel helpless when solving math problems”
Excessive demand	Three items aim to capture the extent of feeling overwhelmed by math classes.	Assessment for commercial trainees^249^	“In math class, the amount of content is too much”
Math self-concept	Five items ask about students’ beliefs regarding their math abilities and skills.	SCMAT survey, used in the PISA 2012 assessment^248^	“I have always been convinced that math is one of my best subjects”
Interest in math	Four items assess interest in math as a trait.	INTMAT survey, used in the PISA 2012 assessment^248^	“I like math books”
Cognitive and behavioral engagement	Nine items on cognitive engagement in math classes measure the extent to which students use deep learning and appropriate cognitive strategies to improve their understanding. Eight items estimate behavioral engagement in math classes by assessing involvement and active participation in math class.	Math and Science Engagement Scales^250^	“I try to understand my mistakes when I get something wrong” “I put effort into learning math”

The motivational-affective characteristics were assessed using self-report scales. The response options for all scales assessing the motivational-affective characteristics were based on a Likert scale (1 = “*rarely*”, 2 = *“sometimes”*, 3 = *“often”*, 4 = “*mostly*” or 1 = ”*strongly disagree*”, 2 = *“slightly disagree”*, 3 = *“slightly agree”*, 4 = “*strongly agree*”).

#### Fractions on number line knowledge

In the present study, we define (prior) math knowledge as the ability to represent fractions using the number line, which combines conceptual and procedural knowledge^197^ and is, thus, a valid representation of math knowledge^198^. Specifically, we refer to it as (prior) fractions on number line knowledge. Math educators developed the pre- and post-tests to assess (prior) fractions on number line knowledge based on their classroom teaching experience and existing research. In all items, fractions had to be placed on a number line. Items varied in the presence or absence of difficulty-generating factors^191^, such as the denominator fitting to the unit of the number line (or not), the length of the number line being 1 (or not), and subdivisions being present (or not).

Before the start of the intervention, a pre-test with eight items assessed students’ prior fractions on number line knowledge. After each block, a small post-test with six to eight items assessed the fractions on number line knowledge to assess the effects of the interventions. The performance value for the analyses was then calculated from the mean value of all four post-tests. We only calculated scores for students who completed all four tests.

In all pre- and post-tests, smaller numbers indicate higher knowledge and vice versa since the results indicate the deviation from the target fraction on the number line. Thus, we evaluate how far a student’s placement of a fraction on the number line differs from the correct target location. This measure provides the sincerest evaluation of a student’s performance (i.e., fraction on the number line knowledge). Without comparing the student’s placement to the correct position on the number line, the absolute value of their placement alone would offer no meaningful insight into their performance. Accordingly, a score asymptotically approaching zero would reflect a perfect performance.

### Statistical analysis

We applied latent profile analyses (LPA) to answer our first research question and understand the typical profiles of students from different SES backgrounds. Prior fractions on number line knowledge, the three cognitive and the five motivational-affective characteristics were considered indicator variables that define the profiles.

For each profile solution, we identified the Bayesian Information Criterion (BIC)^199^ the sample-size adjusted Bayesian Information Criterion (aBIC)^200,201^, and Akaike’s Information Criterion (AIC)^202^. Moreover, in each case, we considered the entropy, which evaluates the quality of the classification of individuals into different profiles^203^. Ideally, the entropy should be 0.8 or above (i.e., 1 indicates a perfect classification while 0 indicates an uncertain, ambiguous one).

After deciding on the best-fitting profile solution, we included SES as a predictor, which allowed us to investigate the influence of SES on the probability of being assigned to the different profiles, as we were particularly interested in students from lower SES backgrounds. Afterward, we compared the average SES of the profiles that had a significant relationship with SES with the nationwide average SES of students as reported in the recent PISA results^204^. Only profiles with an average ISEI below 51.9 are considered to have a lower SES and will be the students focused on in the present study. The addition of such a sample-independent measure to the data-driven approach is intended to ensure greater generalizability of our results, as we do not have a representative sample with students from only four schools, and higher-SES students seemed to be slightly overrepresented in our sample.

Afterward, we aimed to answer the second research question by fitting a linear mixed model (LMM). We used the profiles (that included the nine cognitive and motivational-affective characteristics as well as SES), the conditions, and the school type as fixed and the class as random coefficients. The fractions on number line knowledge (aggregated results of the four post-tests) represented the outcome variable. All profiles, including those of students with higher SES, were included in the analysis. However, in our discussion, we focus on the profile where SES had a strong negative influence (i.e., the profile to which students from lower SES backgrounds were most likely assigned) with an average SES below the nationwide average SES^204^.

We report all data exclusions and all measures in the study and are guided by the APA Journal Article Reporting Standards (JARS). The statistical analyses were performed in R^205^ version 4.2.1 and R-Studio^206^ as well as in MPlus^207^ version 1.8.7. To conduct the LMM in R we used the lme4^208^ and the lmerTest^209^ packages. All quantitative statistical analyses were performed using an alpha level of .05. This study’s design and its analysis were not pre-registered. Data and analysis code for this study are available by emailing the corresponding author.

## Results

### Descriptive statistics

The descriptive statistics for all variables used for the LPA across all participants can be found in Table 2, and all intercorrelations between variables in Table 3.

**Table 2:** Means, standard deviation and reliability for the considered variables

					Reliability	
Variable	*M*	*SD*	*n*	Number of items	*α*	*ω*
Cognitive characteristics						
Prior FoNLK	0.16	0.13	275	8 in 4 blocks	.98^a^	.98
Sustained attention	0.07	0.59	314	–	.88^b^	–
General reasoning	4.06	3.92	320		.89	–
Visuo-spatial ability	1.60	1.22	321	5	.44^c^	.45^c^
Motivational-affective characteristics						
Math anxiety	2.14	2.14	321	5	.83	.83
Excessive demand	2.04	2.04	321	3	.75	.76
Math self-concept	2.47	2.47	321	5	.86	.87
Math interest	2.17	2.17	321	4	.84	.84
Engagement	2.88	2.88	321	17	.87	.87
FoNLK	0.101	0.09	283	30 in 4 blocks	.81^a^	.82
SES	55.10	18.20	321	–	–	–

The range of possible scores for sustained attention was between −1 and 1, for general reasoning between 0 and 16 and for visual-spatial ability between 0 and 5.

The ranges for all motivational-affective characteristics are between 1 and 4 (as the values of the respective Likert scales).

As for Prior FoNLK and FoNLK, the scores indicate the deviation from the target position, scores had to be = 0 (perfect placement) or >0. For Prior FoNLK the scores ranged from 0 to 0.44 and for FoNLK from 0 to 0.56.

*M* mean, *SD* standard deviation, *FoNLK* Fractions on number line knowledge

^a^To estimate the reliability of the sustained attention, we estimated the split-half reliability. The items were divided after half of the time (90 s) to prevent overestimation.

^b^To estimate the reliability of the Prior FoNLK and FoNLK, we used the results of the four pre-tests and the four post-tests, respectively.

^c^When looking at the low coefficients for the visual-spatial ability tests, note that for speeded tests like this conventional reliability estimates might not accurately reflect reliability. Instead, parallel forms should be used^251^.

**Table 3:** Correlations of the cognitive (1–4) and motivational-affective characteristics (5–9)

	1	2	3	4	5	6	7	8	9
Cognitive characteristics									
Prior FoNLK (1)	–								
Sustained attention (2)	−.24***	–							
General reasoning (3)	−.53***	.39***	–						
Visuo-spatial ability (4)	−.30***	.25***	.42***	–					
Motivational-affective characteristics									
Math anxiety (5)	.31***	−.23***	−.30***	−.29***	–				
Excessive demand (6)	.25***	−.11*	−.24***	−.18**	.62***	–			
Math self-concept (7)	−.34***	.23*	−.39***	.30***	−.66***	−.59***	–		
Interest in math (8)	−.04	.12*	.14*	.08	−.26***	−.37***	.52***	–	
Engagement (9)	−.26***	.27***	.32***	.20***	−.5***	−.53***	.64***	.60***	**–**
SES	−.35***	.29***	.35***	.26***	−.23***	−.21***	.22***	−.01	.26***

*FoNLK* Fraction on number line knowledge.

*p < .05; **p < .01; ***p < .001.

To provide more background information, we report the SES across the different types of schools. The SES differs between students in the different school tracks with *M* = 63.7 (*SD* = 14.6) at academic track schools and *M* = 42.7 (*SD* = 15.7) at non-academic track schools. This difference is statistically significant (*t*(319) = −12.30, *p* = −12.30, *p* < 0.001).

### Latent profile analyses to identify profiles of students from lower SES backgrounds

#### General identification of profiles

**Fig. 2:**
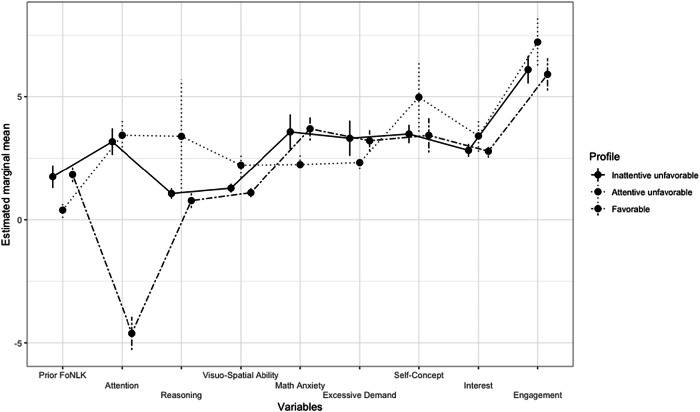
Estimated marginal means on the nine cognitive and motivational-affective characteristics for the three profiles in the final solution. The error bars represent the 95% confidence intervals. Prior FoNLK Prior fractions on number line knowledge.

To answer our first research question and identify profiles of students from lower SES backgrounds, we conducted an LPA. First, keeping the relatively small sample in mind, a model with only two profiles was estimated. Step by step, we estimated models with additional profiles to identify the best-fitting model based on the Information Criteria (IC). The lowest IC values indicate the best fit^210^. We created and compared LPAs with two to six profiles (see Table 4). Entropy was above the required value of 0.8 for all profile solutions; the value was highest for the three-profile model. The lowest and thus best values for AIC, BIC, and aBIC were shown for the five-profile model, followed by the six-profile model and then the three-profile model. Looking at the corresponding profile plots, we discovered relevant disadvantages of these two solutions. For most variables, the five-profile solution indicated two similar strands of profiles, comprising two and three profiles, respectively, and thus actually representing two discriminatory profiles. The six-profile model indicated two profiles with membership counts of zero and approaching zero; eventually, only indicating four relevant profiles. In contrast, in the three-profile model (Fig. 2), at least one profile was clearly distinguishable from the other two which also differed in a practically meaningful way. Thus, we referred to the principle of parsimony and aspects of interpretability^211^ and decided to use the three-profile model.

**Table 4:** Information criteria and entropy for solutions with 2–6 profiles

Number of profiles	AIC	BIC	aBIC	Entropy
2	5845.07	5954.45	5862.46	0.84
3	5508.62	5659.49	5532.61	**0.99**
4	5528.43	5720.78	5559.01	0.85
5	**5204.94**	**5438.77**	**5242.12**	0.89
6	5340.51	5615.82	5384.28	0.93

Best values according to information criteria are in bold.

The fit of this final three-profile model deteriorated when within-profile correlations among the nine indicator variables were set free. This suggests adherence to the assumption of local independence^212^, indicating that the indicator variables seem uncorrelated within the profiles. Moreover, the model fit did not improve when we estimated the variances for each indicator variable. So, aligned with the assumption of homogeneity of variances and the principles of parsimony, we constrained them equally across profiles.

#### Description of the final three-profile solution

We named the three profiles according to the estimated values of the variables and their relation to learning, meaning those combinations in profiles that seem disadvantageous for learning and academic performance are called unfavorable. In contrast, the one that seems advantageous is called favorable: Inattentive unfavorable, attentive unfavorable, favorable. Figure 2 and Table 5 show the estimated values of the nine cognitive and motivational-affective characteristics for the three profiles. Additionally, Table 5 displays the number of students assigned to each profile, as the LPA provides a probabilistic estimate of which profile each student is most likely to belong to.

**Table 5:** Membership counts [and proportions] and estimates for the cognitive and motivational-affective characteristics for the final three profiles

		Cognitive characteristics				Motivational-affective characteristics				
Profile	*N* [Proportions]	Prior FoNLK [95% CI]	Sustained attention [95% CI]	General reasoning [95% CI]	Visuo-spatial abilities [95% CI]	Math anxiety [95% CI]	Excessive demand [95% CI]	Math self-concept [95% CI]	Interest in math [95% CI]	Cognitive and behavioral engagement [95% CI]
Inattentive unfavorable	116 [23.36]	1.83 [1.53, 2.14]	−4.62 [−5.29, −3.95]	0.78 [0.48, 1.07]	1.10 [0.92, 1.28]	3.7 [3.23, 4.17]	3.21 [2.79, 3.64]	3.43 [2.74, 4.13]	2.79 [2.53, 3.05]	5.90 [5.25, 6.56]
Attentive unfavorable	130 [36.14]	1.75 [1.29, 2.20]	3.18 [2.63, 3.72]	1.07 [0.85, 1.28]	1.28 [1.10, 1.46]	3.57 [2.87, 4.28]	3.32 [2.60, 4.03]	3.49 [3.12, 3.86]	2.83 [2.56, 3.09]	6.10 [5.54, 6.65]
Favorable	75 [40.5]	0.39 [0.08, 0.70]	3.44 [2.78, 4.10]	3.39 [2.78, 4.10]	2.22 [1.71, 2.73]	2.25 [2.23, 2.70]	2.33 [2.07, 2.59]	4.98 [3.59, 6.37]	3.41 [2.77, 4.04]	7.23 [6.27, 8.18]

Memberships are based on the most likely membership pattern.

Higher numbers for Prior FoNLK indicate lower knowledge, since the result indicates the deviation from target fraction on the number line.

*Prior FoNLK* Prior fraction on number line knowledge.

Employing the Wald test of parameter constraints, we could reveal whether the profiles differ significantly. The chi-square values (Table 6) indicated strong similarities between the attentive unfavorable and inattentive unfavorable profiles. Significant disparities were only found for sustained attention. Conversely, the inattentive unfavorable profile differs significantly from the favorable profile across all estimated variable values. Similarly, the attentive unfavorable profile exhibited significant differences from the favorable profile in all variables except for sustained attention and interest in math.

**Table 6:** Comparisons between the nine indicator variables estimated for the three profiles

Profiles				Cognitive characteristics						Motivational-affective characteristics									
		Prior FoNLK		Sustained attention		General reasoning skills		Visuo-spatial abilities		Math anxiety		Excessive demand		Math self-concept		Interest in math		Cognitive and behavioral engagement	
		*χ*^2^	*p*	*χ*^2^	*p*	*χ*^2^	*p*	*χ*^2^	*p*	*χ*^*2*^	*p*	*χ*^*2*^	*p*	*χ*^*2*^	*p*	*χ*^*2*^	*p*	*χ*^*2*^	*p*
1	2	0.20	.654	2587.18	<.0001	2.64	.105	1.97	.160	0.32	.569	0.21	.645	0.05	.826	0.04	.843	0.92	.337
1	3	48.35	<.001	1463.00	<.001	24.96	<.001	24.99	<.001	37.08	<.001	15.26	<.001	34.67	<.001	6.58	.010	55.11	<.001
2	3	21.24	<.001	1.00	.317	12.89	<.001	16.57	<.001	11.16	<.001	7.47	.006	11.56	<.001	2.845	.091	18.322	<.001

Profile 1: Inattentive unfavorable, Profile 2: Attentive unfavorable, Profile 3: Favorable.

The comparisons between the parameters are based on the chi-square value of the Wald test of parameter constraints.

*FoNLK* Fraction on number line knowledge.

#### Association of profiles with SES

We included SES as a predictor in the three-profile model to examine which profiles were particularly common among students with lower SES. Using the favorable profile as the baseline, we could see that SES had a significant negative influence on the inattentive unfavorable profile with an estimate of −0.07 (*SE* = 0.01*, p* < .001) and the attentive unfavorable profile with an estimate of −0.05 (*SE* = 0.01, *p* < .001), indicating that students from lower SES backgrounds were more likely to be assigned to these two profiles. An exploratory ANOVA supported this finding by revealing a significant effect of SES on profile (F(2, 198) = 41.2, *p* < .001), indicating significant SES mean differences between the three profiles. See Supplementary Table 1 for the results of the post-hoc test. Descriptive statistics show that the inattentive unfavorable profile had an average SES of *M* = 47.5 (*SD* = 16.7), the attentive unfavorable profile of *M* = 54.7 (*SD* = 17.8), and the favorable profile of *M* = 67.7 (*SD* = 14).

Given the comparison to nationwide data on students’ average SES^213^, we consider the 116 students in the inattentive unfavorable profile as students from lower SES backgrounds, compared to students in the attentive unfavorable profile with a medium SES, and those in the favorable profile with a higher SES.

#### Description of profiles

##### Inattentive unfavorable profile

The students in this profile exhibited the most unfavorable values compared to the other two profiles across all cognitive and motivational-affective characteristics. As all these characteristics interact with academic performance, the combination of lower cognitive characteristics and disadvantageous scores on the motivational-affective characteristics suggests that the students in this profile are less likely to excel in math than their peers in the other profiles. Indeed, the Games–Howell post-hoc test indicated a significant mean difference of 0.16 (*p* < .001) in the prior fraction on number line knowledge compared to students in the favorable profile. Their post-test results indicated their fraction on number line knowledge also differed significantly from students in the attentive unfavorable profile (mean difference: 0.62*, p* < .001) and in the favorable profile (mean difference: 1.23*, p* < .001). See Supplementary Table 2 for descriptive results and ANOVA outcomes. Therefore, this profile can be generally described as unfavorable regarding learning prerequisites. The particularly strong difference in attention scores to students in the attentive unfavorable profile guided the naming of the profile.

Of the students assigned to this profile, 29.31% (*n* = 34) attended an academic-track school, and 70.69% (*n* = 82) a non-academic-track school.

##### Attentive unfavorable profile

Students in this profile were similar to students in the inattentive unfavorable profile across most characteristics. Even though they scored slightly better on all characteristics, there were no significant differences. However, students in the attentive unfavorable profile exhibited a better capacity to sustain attention. Despite the relevant role of attention for learning and academic success, this profile can still be described as disadvantaged regarding academic performance. The Games–Howell post-hoc test exhibited significant differences to students in the favorable profile in both the prior fraction on number line knowledge (mean difference: 0.16*, p* < .001) and the (post-test) fraction on number line knowledge (mean difference: 0.60*, p* < .001).

Of the students assigned to this profile, 63.85% (*n* = 83) attended an academic-track school, and 36.15% (*n* = 47) a non-academic-track school.

##### Favorable profile

In contrast to the other two profiles, students in this profile showed favorable scores across all characteristics. The combination of these variables constitutes an advantageous foundation for academic achievement.

Of the students assigned to this profile, 97.33% (*n* = 73) attended an academic-track school, and 2.67% (*n* = 2) attended a non-academic-track school.

### Linear mixed models to examine beneficial scaffolds for students from lower SES backgrounds

By fitting an LMM, we examined how different scaffolds might foster students’ fraction understanding. We were particularly interested in how it affected the performance of students in the inattentive unfavorable profile; yet the analysis also allowed us to contrast students in the inattentive unfavorable profile with students in the other profiles, hence providing reference points.

Table 7 displays the distribution among participants assigned to the three profiles to the four conditions.

**Table 7:** Distribution of students to the different conditions

Condition	Inattentive unfavorable	Attentive unfavorable	Favorable	Total
Explanatory feedback	24	40	21	85
Adaptive task difficulty	32	35	17	84
Dynamic visualization	29	33	18	80
Control	31	22	19	72

As the data for the fractions on number line knowledge (post-test) were not normally distributed, we logarithmized them. All other assumptions were tested and fulfilled. To predict the post-test performance, we fitted an LMM with condition (control group as baseline), profile (favorable profile as baseline), and school type (academic-track school as baseline). Additionally, we included class as a random effect to account for the hierarchical data structure and potential nested random effects.

Both the model’s total explanatory power (conditional *R*^2^ = 0.53), and the part related to the fixed effects alone (*R*^2^ = 0.52) were substantial. We found significantly positive effects of the two unfavorable profiles, indicating that an affiliation with these profiles might be detrimental to students’ performance. Moreover, we found a significantly negative effect of the academic-track school, indicating a better performance for students attending this type of school. The effects for neither of the interventions (explanatory feedback, adaptive task difficulty, dynamic visualizations) were significant.

The interaction effect between the adaptive task difficulty condition and the inattentive unfavorable profile was significant and negative, suggesting that students assigned to this profile showed significantly higher performance on the fraction on number line knowledge test in this condition compared to the control condition. Additionally, we found a significant interaction effect between the adaptive task difficulty condition and the school type, indicating that students’ performance at non-academic-track schools was significantly higher if they were in this condition compared to the control condition. All results of the LMM can be found in Table 8.

**Table 8:** Results from the linear mixed model showing main and interaction effects of profiles and condition on fraction on number line knowledge

Random effects	Variance	*SD*				
Class	0.01	0.08				
Residual	0.43	0.66				

Fixed Effects	*β*	*SE*	*df*	CI	*t*	*p*
**Intercept**	**−2.76**	**0.26**	**246.17**	**[−3.27, −2.26]**	**−10.80**	**<.001**
Explanatory feedback (C1)	−0.04	0.34	264.15	[−0.69, 0.62]	−0.11	.912
Adaptive task difficulty (C2)	0.62	0.36	266.09	[−0.06, 1.34]	1.72	.090
Dynamic visualizations (C3)	0.21	0.35	266.37	[−0.44, 0.91]	0.62	.558
**Inattentive unfavorable (P1)**	**1.08**	**0.23**	**265.70**	**[0.65, 1.54]**	**4.80**	**<.001**
**Attentive unfavorable (P2)**	**0.69**	**0.23**	**266.37**	**[0.26, 1.14]**	**3.02**	**.003**
**School type**	**−0.74**	**0.21**	**174.05**	**[−1.13, −0.34]**	**−3.58**	**<.001**
C1:P1	−0.11	0.34	264.26	[−0.76, 0.53]	−0.33	.744
**C2:P1**	**−0.79**	**0.34**	**266.43**	**[−1.48, −0.14]**	**−2.30**	**.022**
C3:P1	−0.28	0.32	266.19	[−0.93, 0.33]	−0.87	.387
C1:P2	0.05	0.30	267.00	[−0.52, 0.62]	0.17	.863
C2:P2	−0.13	0.31	266.80	[−0.74, 0.45]	−0.42	.676
C3:P2	−0.25	0.31	262.36	[−0.86, 0.33]	−0.80	.422
C1:NA-Track	−0.08	0.28	261.38	[−0.61, 0.45]	−0.30	.766
**C2: NA-Track**	**−0.62**	**0.29**	**262.03**	**[−1.17, -0.07]**	**−2.16**	**.031**
C3:NA-Track	−0.34	0.28	265.34	[−0.87, 0.18]	−1.24	.217

Significant results are in bold.

Beta-weights represent the standardized regression weights.

*NA-Track* Non-academic track school.

**Fig. 3:**
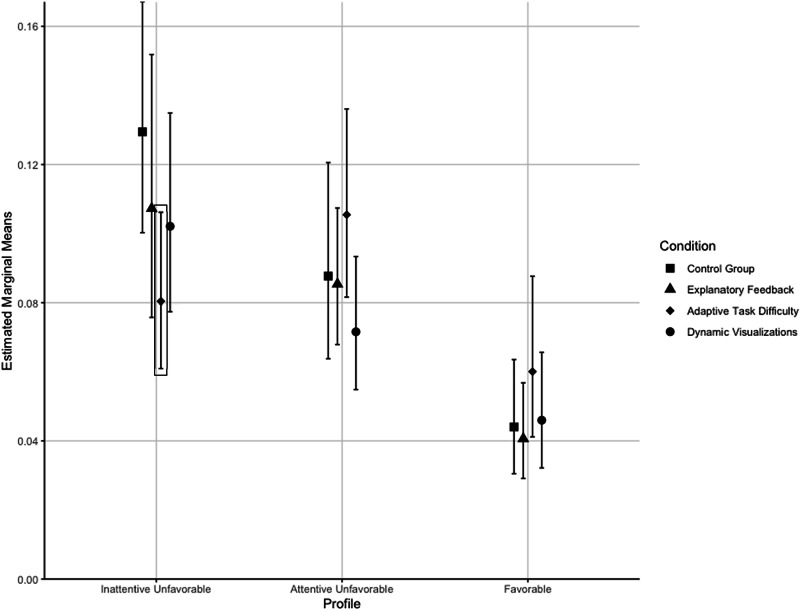
Visualization of estimated means for the fraction on number line knowledge for each profile and condition. Error bars represent 95% confidence intervals.

The estimated means and comparisons of these estimated means are visualized in Fig. 3. Data were exponentialized to the original scale (to reverse the previous logarithmization) before the post-hoc calculations.

The smaller the value of the estimated means for the post-test, the higher the fraction on number line knowledge.

A significant interaction of inattentive unfavorable profile and adaptive task difficulty is pointed out by the black box.

## Discussion

Acknowledging and addressing unfavorable learning prerequisites associated with lower SES early might contribute to preventing negative academic, personal, and societal consequences for the students concerned^214^. Therefore, understanding typical combinations of relevant learning prerequisites of students from lower SES backgrounds is essential to offer them appropriate support. After identifying such profiles, the present research intended to investigate whether adaptive task difficulty classified as a motivational scaffold and explanatory feedback and dynamic visualizations classified as cognitive scaffolds ameliorate math performance in secondary school among students from lower SES backgrounds.

Aligned with our hypotheses, our results suggest that the combination of lower cognitive ability (i.e., lower visuo-spatial abilities, lower non-verbal reasoning skills, and lower sustained attention), heightened math anxiety and excessive demand, and reduced self-concept, interest in math, and engagement are linked to lower SES. The SES in the inattentive unfavorable profile, with an average ISEI of 47.5, was below the German average SES of 51.9, as reported in the recent PISA results^213^. Even though the analysis with SES as a predictor also indicates a higher likelihood of lower SES students being assigned to the attentive unfavorable profile, their average SES is higher than the average SES in Germany^213^. Despite their SES being significantly lower compared to that of the favorable profile, we do not consider these students to be from a lower SES background. Therefore, we will mostly focus on students in the inattentive unfavorable profile in the discussion.

We assumed that these cognitive and motivational-affective characteristics in lower SES students could be targeted with cognitive and motivational instructional scaffolds. We found that students in the inattentive unfavorable profile, which is most strongly associated with lower SES, particularly benefited from adaptive task difficulty. This type of motivation scaffolding notably improved their math performance, suggesting its potential as an effective intervention for students from lower SES backgrounds. Contrary to our expectations, however, the two cognitive scaffolds did not show any significant effects for students in the inattentive unfavorable profile. As expected and aligned with prior research, we found no significant effects for students in the favorable profile^164^, nor in the inattentive unfavorable profile. Thus, in line with the Aptitude-Treatment-Interaction (ATI) research, we found no universal effects of the instructional scaffolds^215^.

In the following, we first discuss the typical profile of students from lower SES backgrounds and then explain whether and how the three types of cognitive and motivational scaffolds have influenced their fractions on number line knowledge in the context of existing research. Based on the discussion points, we derive implications for future research.

Our person-centered approach aimed to discover different profiles among students from lower SES backgrounds. Although the research question may imply causality, we could not investigate this using the available data and methods. Correspondingly, our results were correlative. Nevertheless, they indicated a relationship between SES and cognitive and motivational-affective characteristics.

Generally, the three profiles exhibited a less pronounced mixture of strengths and weaknesses than we had expected. Thus, the profiles could be divided into favorable and unfavorable regarding learning and academic performance. This is most likely a consequence of our relatively small sample, which allowed only dominant profiles to emerge. While the sample size could bias the profiles, the clarity of these dominant profiles and their relation to SES emphasize that many students with lower SES seem to experience at least some disadvantages on various characteristics simultaneously. Future research should conduct similar analyses with a larger sample to detect less frequently occurring ‘mixed profiles’ to paint a more heterogeneous and nuanced picture of students from lower SES backgrounds.

More specifically, our results revealed a predominantly homogeneous pattern when examining the two unfavorable profiles. Attention was the only distinguishing factor. Considering that the inattentive unfavorable profile is characterized by a below-average SES, this aligns with prior research indicating a consistent trend for these students to have fewer opportunities to develop their cognitive abilities and might establish unfavorable combinations of motivational-affective characteristics. As described in the introduction, we assume these outcomes might stem from adverse home learning environments and a lack of family support, which have the potential to hinder students from lower SES backgrounds from reaching their potential and influence their attitudes about themselves and school^216^. There is evidence that many low-income families might have similar life circumstances, which include, for example, fewer opportunities for cognitive development at home^22,23^, potentially contributing to the observed similarities in visuo-spatial abilities and reasoning skills among students from lower SES backgrounds.

Moreover, lower SES is generally associated with adverse psychological outcomes^217^. These outcomes potentially arise from internal factors, such as students’ experience of poverty-related stress^218^, and external factors, including encountering prejudices and stigma^34,112^. This might elucidate why students in the two unfavorable profiles in our study tend to have a low self-concept in math^115,219^, high math anxiety^134^, lower engagement^102^, and high excessive demand^140^.

The lower interest in math among students in the two unfavorable profiles could be explained by possible prior experiences of failure in math classes^122^, since academic setbacks such as receiving bad grades harm interest^124^. This link between low performance and interest seems to be plausible, as the students in the two unfavorable profiles performed comparatively poorly in the prior fractions on number line knowledge test.

The contrasting sustained attention scores of the two unfavorable profiles are noticeable. While one group of students exhibited comparatively low values, the other group demonstrated attention scores akin to those observed in the favorable profile. A possible explanation for the attention differences might be the school type that students attend. While almost all students in the inattentive unfavorable profile attend a non-academic track school, approximately two-thirds attend an academic track school. Considering the institutional and compositional effects of different types of schools^220^ it seems plausible to assume that the environment at the academic track has positively influenced students’ sustained attention. The student body at academic track schools is predominantly composed of higher-performing students with higher SES^221^. This can be particularly beneficial for lower SES students and positively influence to their performance^222^. However, whether this is an actual causality or, for example, a selection effect because the more attentive students tend to attend the academic track school cannot be clarified based on our data.

As expected, the present study showed that the three types of scaffolding with different scaffolding intentions do not seem to be universally effective. Instead, the present study revealed that specific interventions were advantageous for students with specific profiles. This finding suggests a differential effectiveness, specifically the contingency of instructional support effectiveness on individual factors. As such, the results are aligned with the ATI perspective that proposes that based on their individual learning prerequisites, learners respond differently to different educational interventions^215^. This strand of research has more recently been termed the *Heterogeneity Revolution*^223^, which acknowledges the differences in the effectiveness of interventions across populations and contexts. Consequently, the results indicate the relevance of considering sub-groups when analyzing the effectiveness of educational interventions, such as scaffolding.

More precisely, we found significant benefits of adaptive task difficulty for students in the inattentive unfavorable profile, as evidenced by their fractions on number line knowledge. As a motivational scaffold^113^, we infer that adaptive task difficulty might have particularly addressed the unfavorable motivational-affective characteristics of those students by providing a positive learning experience and fostering feelings of accomplishment and competence^159,163^. Working on tasks they could solve, potentially eliciting feelings of enjoyment and pride, enables better performance^224,225^.

Another advantage of the adaptive task difficulty is the subtlety with which tasks in the digital learning environment are tailored to students’ needs and prerequisites. Unlike overt forms of differentiation based on performance, such as school tracking, adaptive task difficulty prevents students from realizing differences in performance levels among their classmates. This subtlety avoids emotional impairment in the form of feelings of segregation and devaluation^226^, which can harm motivation and academic achievement^227^.

It remains to be investigated why the students in the attentive unfavorable profile, who were very similar to those in the inattentive unfavorable profile, did not benefit equally from adaptive task difficulty. It might be that inattentive students typically only work on a few tasks during the voluntary practice phases, especially if they are too challenging or too easy for them. This might have been the case for students with low attention scores in the control group. Conversely, having tasks adapted to students’ level of performance may have enhanced their motivation and led to prolonged engagement with the exercises. In the data, that might have increased the differences between the adaptive task difficulty condition and the control condition for the students in this profile. In contrast, the more attentive students might naturally complete more tasks during such an exercise period, increasing the chance of encountering tasks that fit their abilities and keep them motivated. Consequently, there was no significant difference between the adaptive task difficulty condition and the control condition for students in the attentive unfavorable profile. In future research, process data could be used to investigate whether there are differences in the number of tasks completed in the practice phases that could potentially over- or underestimate the differences between the groups. Additionally, motivation could be included as a mediator to test the assumed mechanism of the motivational scaffold. Moreover, it could be insightful to investigate other motivational scaffolds (e.g., motivational feedback) to see whether they have similar positive effects, especially when being more explicit in emphasizing improvement, learning processes, and potential over mistakes and deficits^228^.

Explanatory feedback, classified as an information scaffold, and dynamic visualizations, classified as an information-processing scaffold^113^, showed no significant impact on the students from lower SES backgrounds in the inattentive unfavorable profile. These types of scaffolds require active engagement, and the lack of significant effects suggests an insufficient engagement with and attention to the scaffolds. This would be reasonable, as the students had relatively low scores on engagement and attention. This assumption aligns with a common issue related to scaffolding: Students often ignore the scaffolds due to motivation difficulties or comprehension difficulties^229,230^. Log data with timestamps or eye-tracking/sensor data would allow validation of the assumption in the future by seeing how and how long students engage with the scaffolds.

The results can also be related to students’ metacognitive processes, which we did not consider in the present research, but which play a significant role in the effectiveness of scaffolding^154^.

Moreover, the results raise considerations about the general effectiveness of scaffolds with motivational scaffolding intentions compared to scaffolds with cognitive scaffolding intentions for students from lower SES backgrounds with similar profiles to those in our sample. As cognitive functions are linked to emotional factors, several motivational-affective characteristics, such as a low self-concept, pose challenges to students’ academic performance^110^. Furthermore, negative emotions and unfavorable motivational-affective characteristics might impede the ability to use cognitive scaffolds, potentially explaining the lack of significant effects. For example, students from lower SES backgrounds might lack motivation, which could hinder their engagement with cognitive scaffolds. Instead, strengthening their perspectives on themselves and their attitudes toward school subjects might be pivotal to their learning^23,231,232^. Considering this, motivational scaffolds might be necessary to lay the groundwork before introducing cognitive scaffolds. Future research could explore whether cognitive scaffolds would be more effective for students from lower SES backgrounds with more favorable scores on the motivational-affective characteristics.

Considering the results within the German school system, which tracks students after elementary school based on their performance, our results suggest that school type has an impact on performance beyond the students’ profiles and support the finding that lower SES students seem to benefit from adaptive task difficulty. This is evident in the interaction effect of school type with adaptive task difficulty, in addition to the interaction between the inattentive unfavorable profile and this motivational scaffold. Moreover, we observed that students from the favorable profile almost exclusively attend the academic-track school, while students from the inattentive unfavorable profile mostly attend the non-academic-track school. In Germany, elementary school teachers suggest a type of secondary school for their students. However, this suggestion may not only include performance measures but might also be influenced by SES and migration background^233,234^. The parents usually make the final tracking decision that can depend on their academic aspirations, which are often related to their SES^235^. Past research indicates that the types of schools differ in the composition of students, with more students with higher SES enrolled in academic track schools and more students with lower SES enrolled in non-academic track schools^188,221^, highlighting that the present results are, to some extent, not surprising. This is also reflected in the lower average SES of students at the non-academic track school. With 42.7, it is lower than the German average^213^ and also lower than that of the students in the inattentive unfavorable profile.

There are multi-causal patterns of disadvantage in non-academic track schools, including, in particular, the institutional effects of the different learning environments^236^ and the differing student compositions^220,237^. Overall, students at non-academic track schools might receive less support and stimulation than their peers at academic track schools^238^. A closer look at the underlying causes and explanatory mechanisms of why adaptivity seems to benefit students at non-academic track schools should be the focus of future research.

It is relevant for teachers and other educators to be aware of the different prerequisites students bring into the classroom^67^. To strive toward equity in schools, students should not all be treated equally but according to their needs. Understanding these needs allows teachers to help students and create suitable learning and support opportunities. Being particularly sensitive to the challenges students from lower SES backgrounds face might help dispel stereotypes associated with lower SES^112^. Acknowledging why students from lower SES backgrounds might perform worse and that there are ways to counteract this could motivate teachers to invest effort and support them need-based. The present study highlights adaptive task difficulty as an effective motivational scaffold for students from lower SES backgrounds with specific profiles that are disadvantageous for learning. Since this scaffold did not seem to harm students with higher SES in the more favorable profile, implementing adaptive task difficulty in instructional practive is recommended as one potential way to narrow the achievement gap.

As operationalized in the present study, adaptive task difficulty needs appropriate technical hardware and software. However, offering tasks with appropriate difficulty levels and, thus, scaffolding students’ motivation and mastery orientation is possible without the use of technology. We recommend that (prospective) teachers receive training in adaptive competencies to teach adaptively, including offering adaptive task difficulties, even without the appropriate technical facilities^239,240^.

### Limitations and future research

The representativeness of our sample is limited, as we were unable to randomly select participants from all schools. Due to the low response rate from the large number of contacted schools and the inability to obligate any schools to participate, we collected data from all schools that responded positively, potentially resulting in a selective sample. Moreover, an oversampling of lower SES schools would have been desirable for our research questions, but the necessary infrastructure is lacking in Germany.

Considering the short intervals between the intervention and the four post-tests, as well as the lack of other measurement points, we can only observe immediate effects. Future research should incorporate follow-up assessments to get insights into the long-term effects of instructional scaffolding on math performance.

Moreover, the cognitive and motivational-affective characteristics were solely assessed as traits, and no changes were measured over time. In the future, we need to include pre- and post-tests of states to examine possible changes in these characteristics due to scaffolding. Employing repeated measures and focusing on state assessment could elucidate the mechanisms by which different scaffoldings types affect students’ performance and open the black box of the effectiveness of different scaffolding types on math performance.

Additionally, we assessed students’ SES only via their parents’ occupations, which were converted into ISCO-88 and ISEI codes. While this is a common procedure in educational or psychological studies and even in global large-scale studies like PISA^196^, we are aware that SES is a more holistic measure that is influenced by more factors than just parental occupation. The most common variables to assess are parental education, occupation, and income. In addition, other aspects such as cultural dimensions (e.g., the number of books at home) or possessions are often considered^195^. When assessing only one of these variables, we, therefore, do not comprehensively cover what SES encompasses and only approximate the definition through our measurement.

Furthermore, the results suggest a potential mismatch between the test instruments and the target group. It is striking that, in contrast to all other variables, students in the attentive unfavorable profile performed well on the sustained attention test. Notably, attention was measured using a playful instrument that differed significantly from the classic cognitive measures and self-report questionnaires used to assess the other characteristics. These standard tests were possibly not suited to capture students’ abilities entirely. Past research has shown that differences in SES and ethnic background explain significant parts of the variance in test scores, indicating that such standardized knowledge tests might disadvantage marginalized groups^241^. For example, some tasks appears more complex for students from lower SES backgrounds^242^. Thus, the lower scores of students in the two unfavorable profiles on the cognitive characteristics do not necessarily provide evidence of impaired cognitive resources or low prior fractions on number line knowledge. Additionally, assessing motivational-affective characteristics via self-reports might have been too challenging (and tedious) for students that age, particularly those with lower SES^243^. In addition, language barriers may have influenced the results, as the questionnaires are more challenging for non-native speakers to complete, who are reported to be more common among students from lower SES backgrounds^244^. Other aspects, such as the time pressure or the need to concentrate unusually long, might have further impaired the results, leading to an underestimation of the students’ true cognitive and motivational-affective characteristics.

In future studies, we should ensure inclusive assessment tools that suit the target group. Instruments, such as the attention test, which allow cognitive and motivational-affective characteristics to be assessed playfully and enjoyably, would avoid potential disadvantages and provide a more precise assessment.

## Conclusion

The results of the present study pointed out that students from lower SES backgrounds were more likely associated with a rather unfavorable profile regarding cognitive and motivational-affective characteristics compared to their peers with higher SES. Empowering and supporting these students, who may face more challenges in the educational system, is crucial for bridging the achievement gap.

Therefore, the study presented adaptive task difficulty as a motivational scaffold to foster students’ performance in fraction tasks in the inattentive unfavorable profile. Overall, this finding suggests that instruction should consider students’ individual differences and provide tasks that suit each student’s performance level. This may be one step towards educational equity.

## Supplementary information

Supplementary information

## Data Availability

The dataset used and analyzed during the current study is available from the correspondingauthor upon reasonable request.

## Code availability

The underlying code for this study is available from the corresponding author upon reasonable request.
