# Supplementary material for: Unlocking math potential in students from lower SES backgrounds – using instructional scaffolds to improve performance
Source: NPJ Sci Learn. 2025 Sep 23;10:66. doi: 10.1038/s41539-025-00358-7 (PMC12457687; doi:10.1038/s41539-025-00358-7)
Supplement: Supplementary file 1 — Supplementary information [file 41539_2025_358_MOESM1_ESM.pdf]

Supplementary Information

Supplementary Figure 1

Screenshot for Introductory Explanations in E-Textbook on Fractions on the Numberline

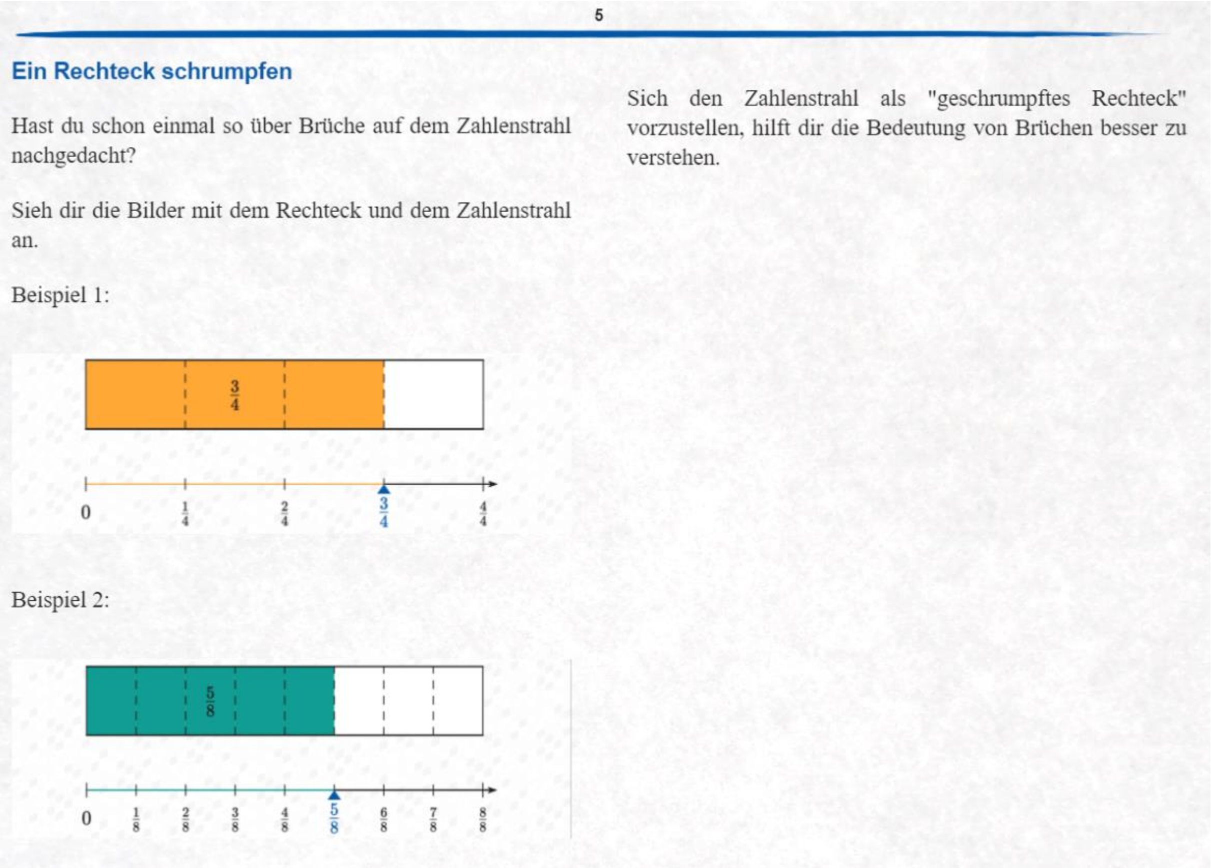
5

Ein Rechteck schrumpfen

Hast du schon einmal so über Brüche auf dem Zahlenstrahl nachgedacht?

Sieh dir die Bilder mit dem Rechteck und dem Zahlenstrahl an.

Beispiel 1:

Beispiel 2:

Sich den Zahlenstrahl als "geschrumpftes Rechteck" vorzustellen, hilft dir die Bedeutung von Brüchen besser zu verstehen.

Note. Translation header: Shrinking a rectangle
Translation left side: Have you ever thought about the bridge on the number line like this? Look at the pictures with the rectangle and the number line.
Translation right side: Imagining the number line as a “shrunken rectangle” helps you to better understand the meaning of fractions.

Supplementary Figure 2

Screenshot of Dynamic Visualization

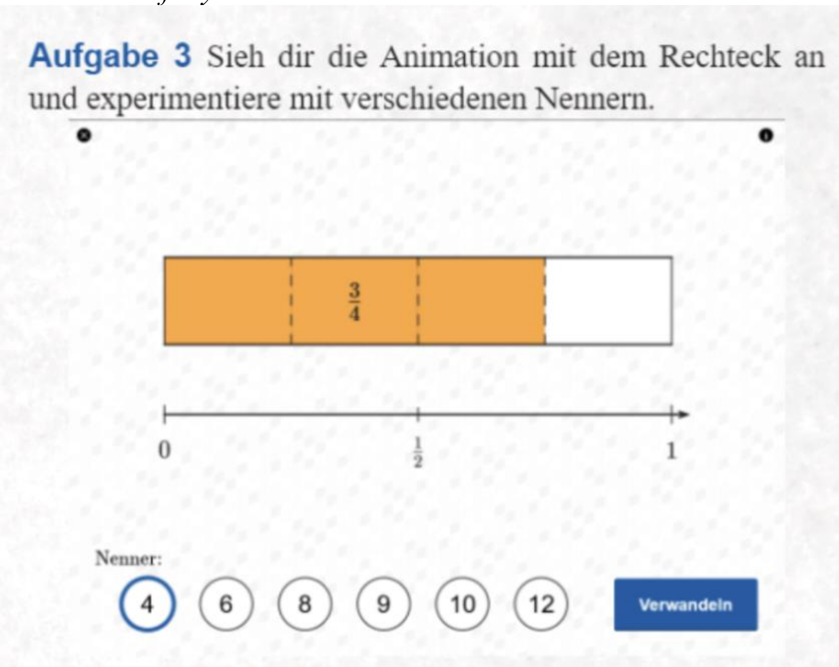

Note. Translation header: Look at the animation with the rectangle and experiment with different denominators.

Translation button on the lower right-hand corner: Transform

Students could alter the visualization by choosing different denominators.

Supplementary Figure 3

Screenshot of Open Answer Reflection Question (for all Conditions)

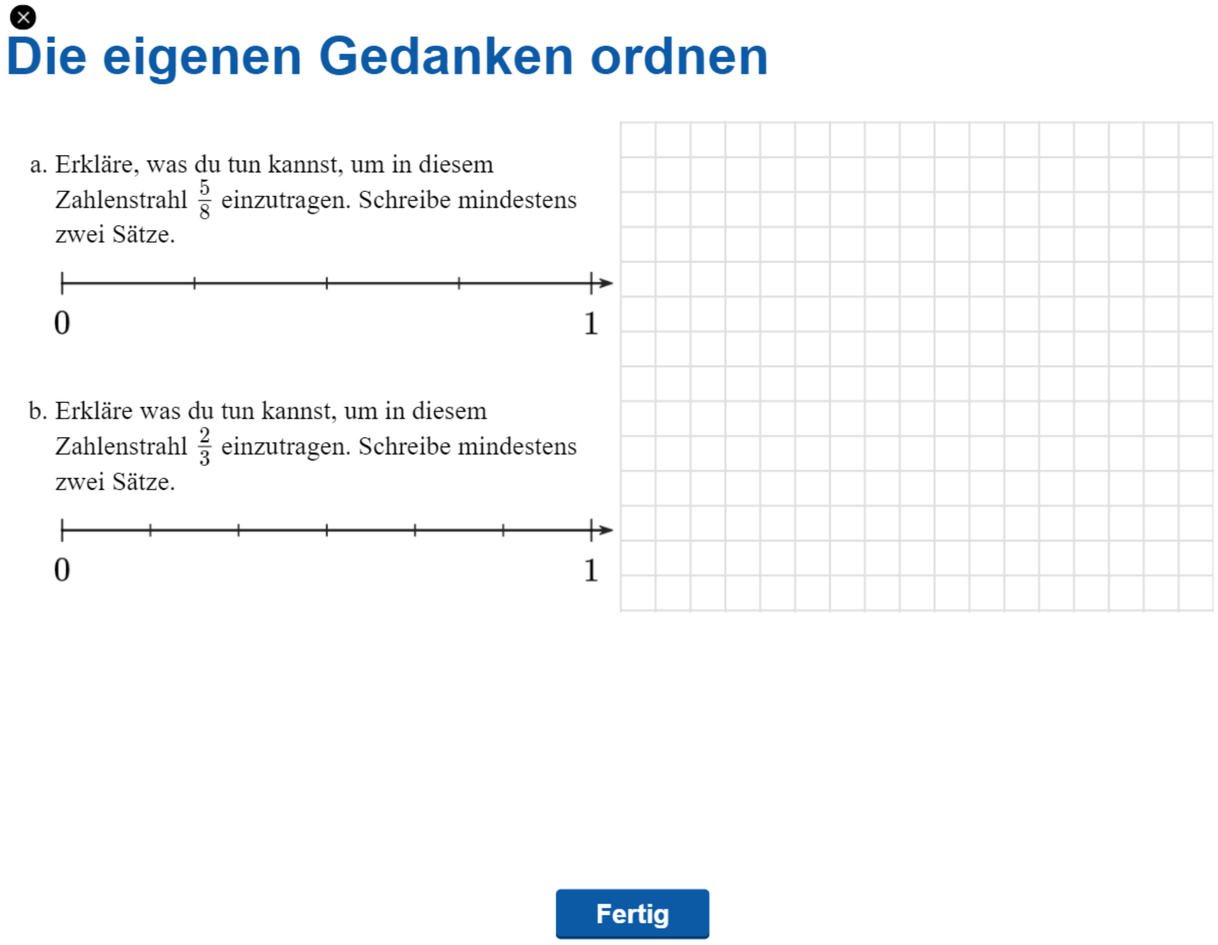

Note. Translation header: Organize your thoughts

Translation reflection question 1: Explain what you can do to enter 5/8 in this number line. Write at least two sentences.

Translation reflection question 2: Explain what you can do to enter 2/3 on this number line. Write at least two sentences.

Supplementary Figure 4

Screenshot of Practice Phase to Place Fractions on the Number Line

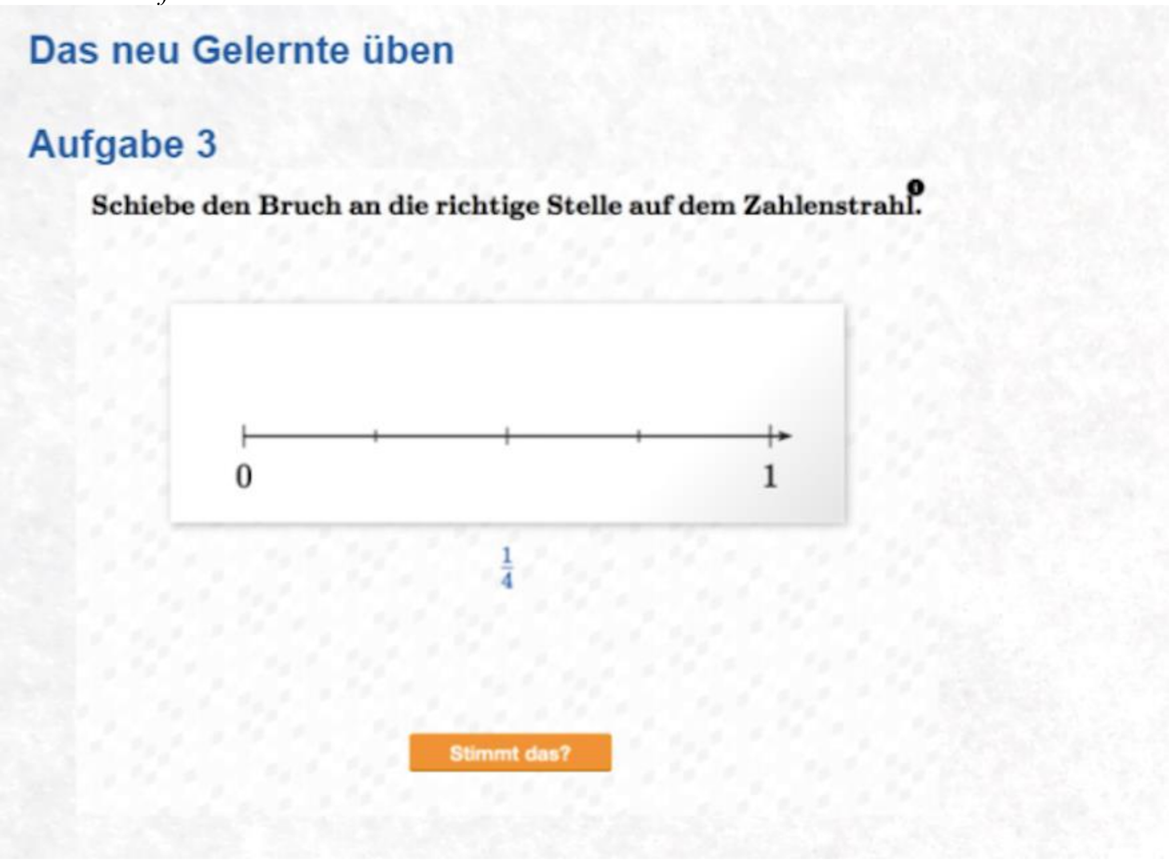

Note. Translation header: Practicing what you have learned

Translation task: Move the fraction to the correct place on the number line.

Supplementary Figure 5

Screenshot of Corrective Feedback during Practice Phase

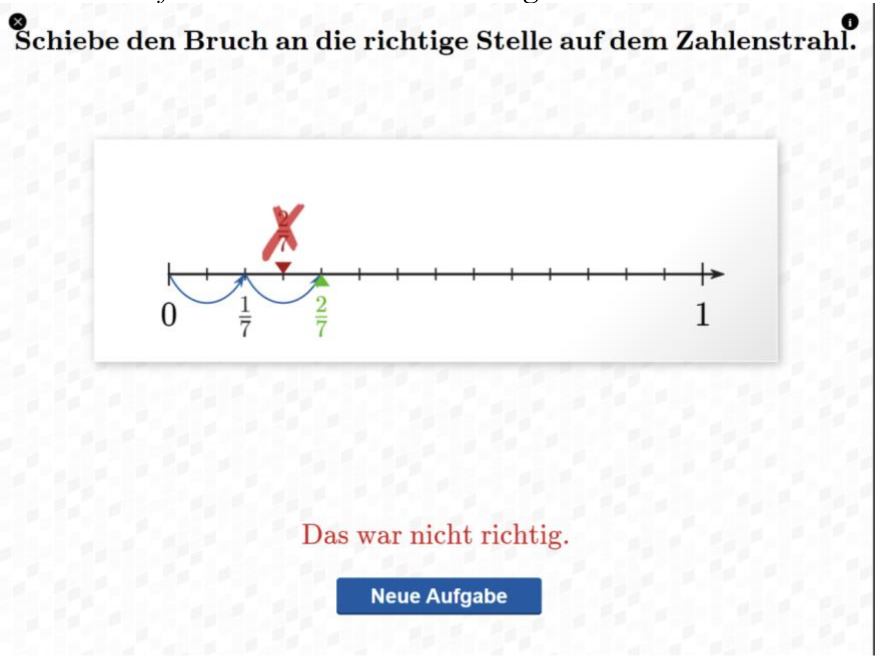

Note. Translation task: Move the fraction to the correct place on the number line.
Translation corrective feedback: That was not correct.
Translation button: New task

Supplementary Figure 6

Screenshot of Explanatory Feedback in Corresponding Condition

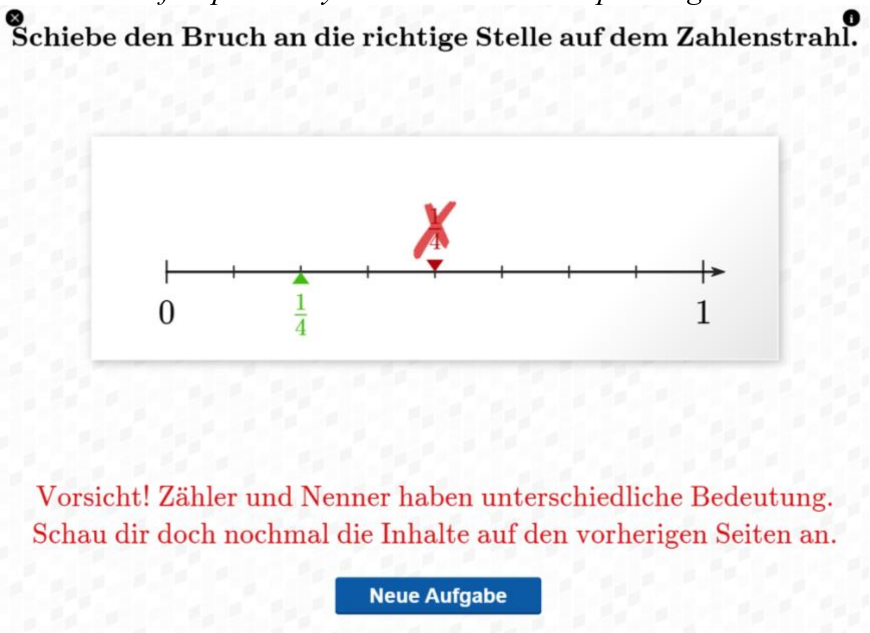

Note. Translation task: Move the fraction to the correct place on the number line.
Translation explanatory feedback: Caution! The numerator and denominator have different meanings. Take another look at the content on the previous pages.
Translation button: New task

Supplementary Figure 7

Sample Item for AST

A

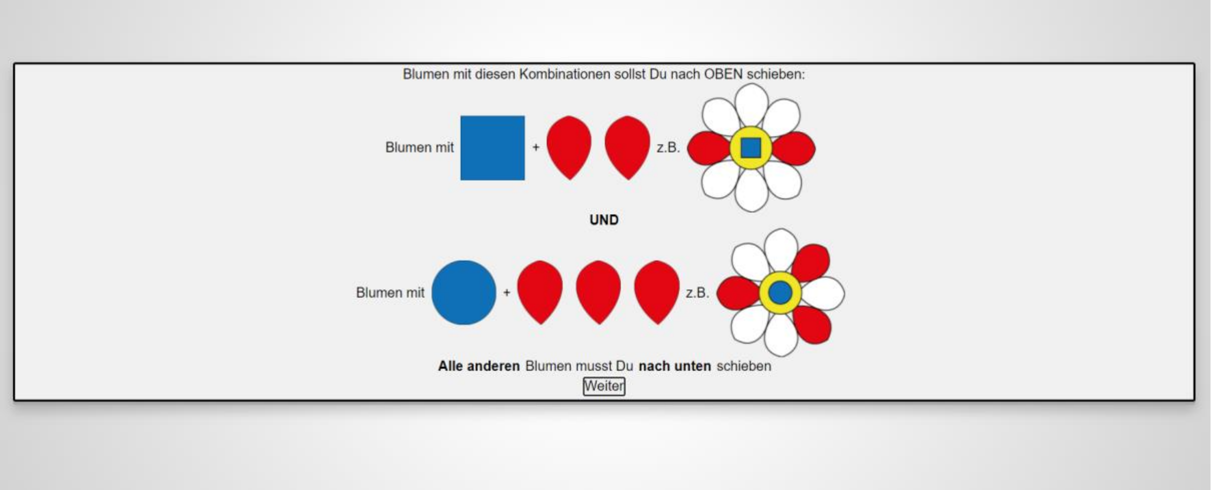

B

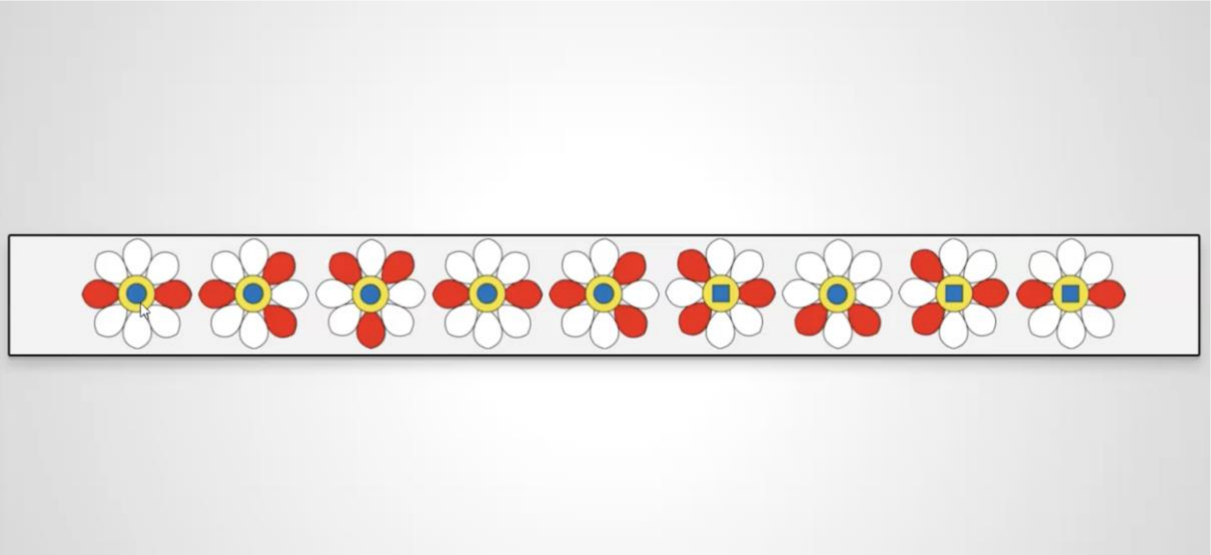

Note. A displays the rules by which students have to sort the flower stimuli. Flowers with a blue square and two red leaves, as well as flowers with a blue circle and three red leaves, need to be sorted up, while all other flowers need to be sorted down. B shows an example of tasks where students need to sort the flowers either up or down as fast as possible according to the rule explained to them before. They have three minutes to complete as many items as possible.

Supplementary Figure 8

Sample Item for DESIGMA Construction-Based Figural Matrices Task

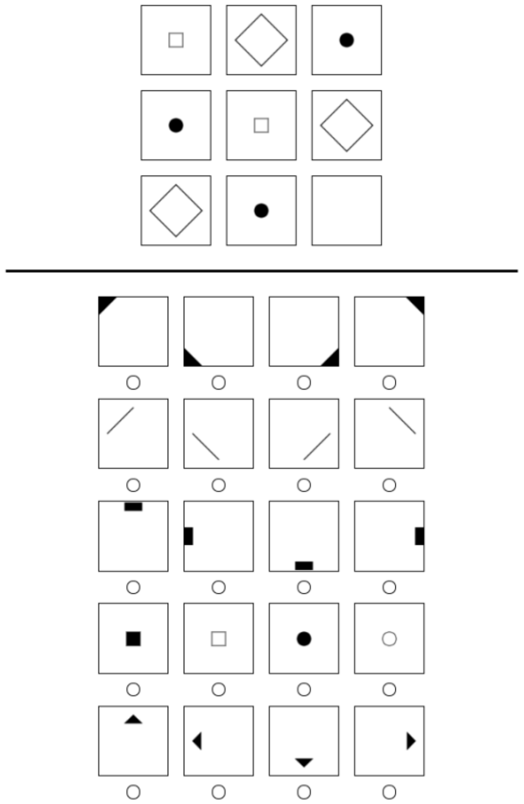

Note. Students must choose all necessary items from below to construct the answer for the ninth field above.

Notably, there are no distractor answers, but the students had to compile the solution without guessing.

Supplementary Figure 9
Sample Item for Paper-Folding-Test

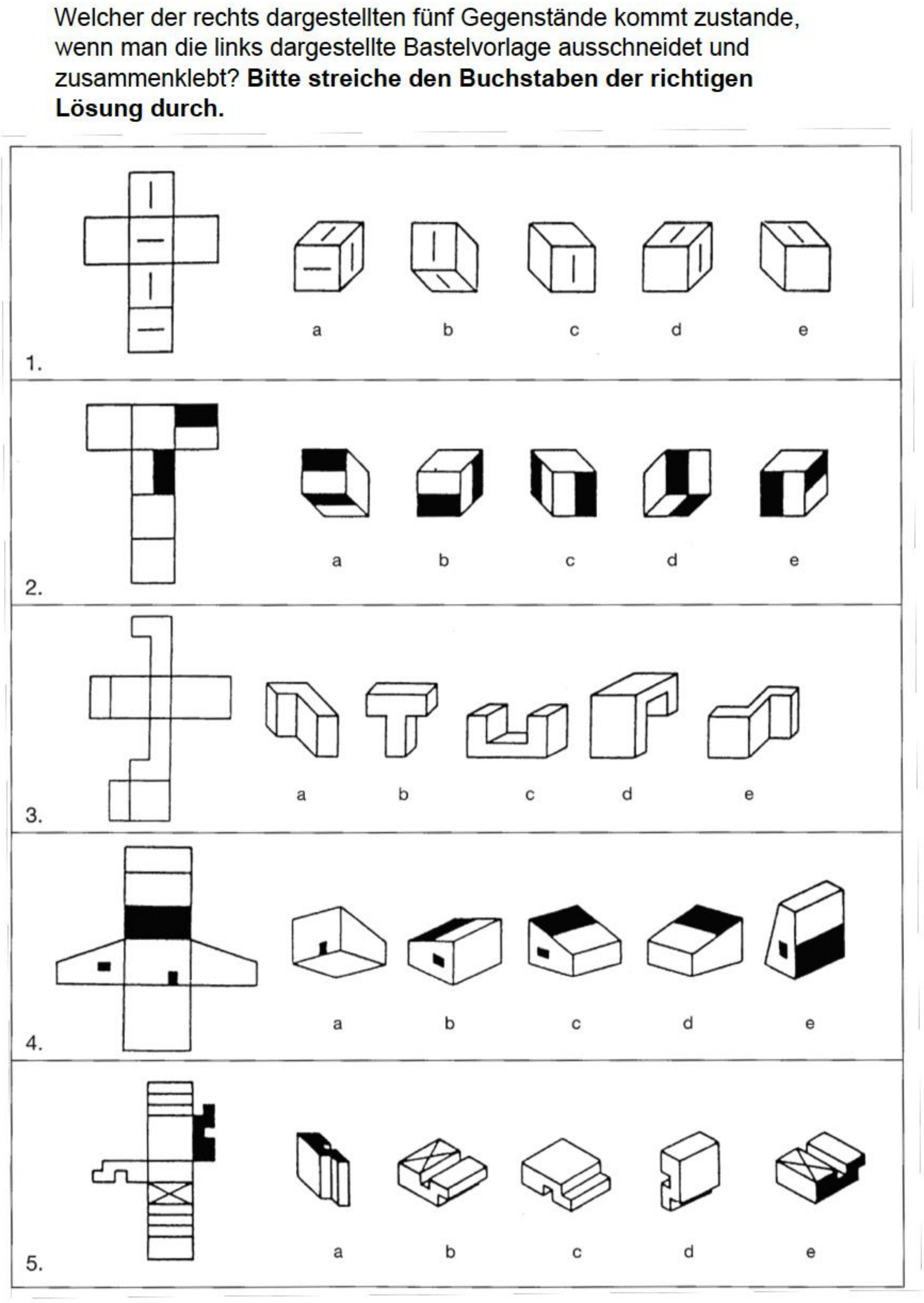

Note. Translation of instructions: Which of the five objects shown on the right will be created if you cut out and glue together the craft template shown on the left? Please mark the letter of the correct solution with a cross.

Supplementary Table 1

Results of the Games-Howell Post-Hoc Test

	1	2	3
Inattentive Unfavorable (1)	__	-7.21**	-20.2***
Attentive Unfavorable (2)		__	-13.0***
Favorable (3)			__

Note. ** $p < .01$, *** $p < .001$

Supplementary Table 2

Means, Standard Deviations and ANOVAS for (Prior) Fraction on Number Line Knowledge

	Inattentive Unfavorable			Attentive Unfavorable			Favorable			*df*	*F*
	n	*M*	*SD*	*n*	*M*	*SD*	*n*	*M*	*SD*		
Prior FoNLK	89	0.21	0.12	112	0.20	0.13	74	0.04	0.06	(2, 175)	97.9***
FoNLK	89	0.14	0.10	114	0.09	0.07	74	0.05	0.03	(2, 153)	82.5***

Note. The higher the value, the worse is the performance.

*** p <.001
